# SARS-Coronavirus Open Reading Frame-8b triggers intracellular stress pathways and activates NLRP3 inflammasomes

**DOI:** 10.1038/s41420-019-0181-7

**Published:** 2019-06-05

**Authors:** Chong-Shan Shi, Neel R. Nabar, Ning-Na Huang, John H. Kehrl

**Affiliations:** B Cell Molecular Immunology Section, Laboratory of Immunoregulation, National Institute of Allergy and Infectious Diseases, National Institutes of Health, Bethesda, MD 20892 USA

**Keywords:** Cell death and immune response, Inflammasome

## Abstract

The SARS (severe acute respiratory syndrome) outbreak was caused by a coronavirus (CoV) named the SARS-CoV. SARS pathology is propagated both by direct cytotoxic effects of the virus and aberrant activation of the innate immune response. Here, we identify several mechanisms by which a SARS-CoV open reading frame (ORF) activates intracellular stress pathways and targets the innate immune response. We show that ORF8b forms insoluble intracellular aggregates dependent on a valine at residue 77. Aggregated ORF8b induces endoplasmic reticulum (ER) stress, lysosomal damage, and subsequent activation of the master regulator of the autophagy and lysosome machinery, Transcription factor EB (TFEB). ORF8b causes cell death in epithelial cells, which is partially rescued by reducing its ability to aggregate. In macrophages, ORF8b robustly activates the NLRP3 inflammasome by providing a potent signal 2 required for activation. Mechanistically, ORF8b interacts directly with the Leucine Rich Repeat domain of NLRP3 and localizes with NLRP3 and ASC in cytosolic dot-like structures. ORF8b triggers cell death consistent with pyroptotic cell death in macrophages. While in those cells lacking NLRP3 accumulating ORF8b cytosolic aggregates cause ER stress, mitochondrial dysfunction, and caspase-independent cell death.

## Introduction

In 2002–2003 the SARS-CoV caused a severe respiratory illness affecting more than 8000 individuals with a mortality rate near 10%^1,2^. The subsequent identification of a large pool of coronaviruses circulating in bats and other animals portended the appearance of other highly pathogenic CoVs^3^, and in 2012 the Middle East Respiratory Syndrome-CoV caused a similar outbreak with a higher mortality rate^4^. Severe SARS-CoV infection manifests clinically as acute lung injury associated with high initial virus titers, macrophage/neutrophil accumulation in the lungs. and elevated proinflammatory serum cytokines (IL-1, IL-18, IL-6, IL-8, and MCP-1)^5–8^. Recent advances have implicated inflammatory monocyte-macrophages (IMMs) in the lungs as critical mediators of SARS-CoV pathology, as a delayed type I interferon response promotes high initial virus titers and aberrant IMM recruitment^9^. Host disease is likely a combination of direct viral damage and consequences of an aberrant immune response promoted by IMMs^9^, underscoring the importance of determining the mechanisms by which the virus targets innate immunity.

The SARS-CoV is an enveloped, positive-strand RNA virus that encodes a set of accessory proteins, several of which target the innate immune response. Open reading frame (ORF) 8a and ORF9b trigger cellular apoptosis; ORF7a activates nuclear factor-κB (NF-κB); ORF3b upregulates the expression of several cytokines and chemokines; ORF6 limits interferon production; ORF3a induces necrotic cell death; and ORF8b induces cellular DNA synthesis and suppresses the expression of the viral envelope protein^10,11^. Recently, we found that ORF9b localizes to mitochondrial membranes and reduces mitochondrial-associated adapter molecule MAVS, severely limiting the interferon response^12^. Of note, ORF8b is of interest as ORF8 encoded a single polypeptide during the early phase of the SARS epidemic, while in the later stages a 29-nucleotide deletion split it into two ORFs, ORF8a and ORF8b. They encode 39- and 84-residue polypeptides respectively. The splitting of ORF8 is thought to confer evolutionary advantage to the virus, and accordingly virus expressing ORF8b is better able to replicate in the presence of interferon^10,13^.

Initially, two studies had difficulty expressing the ORF8b protein, suggesting it may be rapidly degraded in cells^14,15^. However, antibodies raised specifically against the ORF8b protein detect low levels of expression as early as 8 h (h) post infection, while expression is prominent by 24 h^16^. Immunostaining ORF8b after expression in Vero E6 cells localized it to cytosolic punctate vesicle-like structures reminiscent of intracellular aggregates^17^. Here, we investigate the cellular mechanisms by which ORF8b contributes to SARS-CoV pathology. We report that ORF8b forms intracellular aggregates dependent on a valine at residue 77, which contributes to the induction of lysosomal stress, autophagy, and eventual cell death. Given the importance of IMMs in SARS-CoV pathology, the link between intracellular aggregates and inflammasome activation^18^, and the observation that SARS-CoV infected patients have elevated serum levels of IL-1 and IL-18^19^, we tested whether ORF8b might affect inflammasome activation in macrophages and lung epithelial cells. We found that ORF8b triggers robust NLRP3 inflammasome activation and IL-1β release. NLRP3 activation was accompanied by direct binding of ORF8b to the LRR domain of NLRP3. In sum, this study suggests that ORF8b activates cell stress pathways by forming intracellular aggregates and may be important in the aberrant activation of macrophages during SARS-CoV infection.

## Results

### ORF8b forms intracellular aggregates and causes cell death dependent on valine 77

**Fig. 1:**
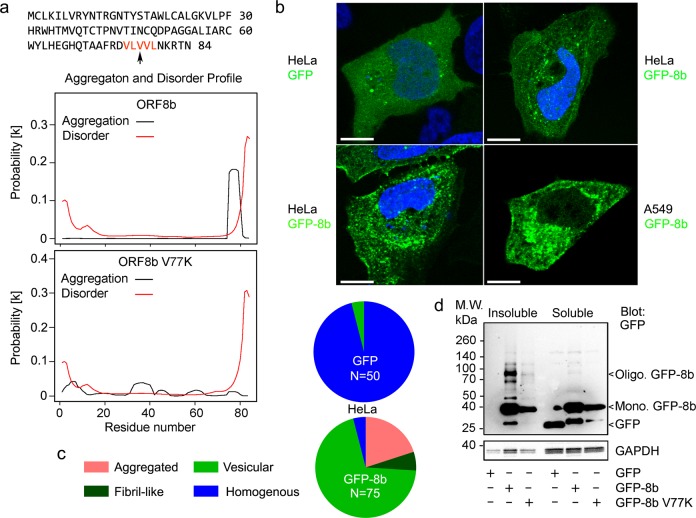
ORF8b forms intracellular protein aggregates whose presence depend on V77. **a** ORF8b amino acid sequence and predicted aggregation and disorder profile. The amino acid motif predicted to be critical for ORF8b aggregation by PASTA 2.0 (protein aggregation prediction server, http://protein.bio.unipd.it/pasta2/) is colored red. Aggregation and disorder profiles are shown for ORF8b and mutant (V77K) ORF8b. **b** Confocal microscopy images of HeLa cells transiently transfected with GFP-8b show intracellular patch and fiber-like protein aggregates. Nuclei are counterstained with DAPI and the scale bar is 10 μm. **c** Quantification of percentage of GFP-positive cells with protein aggregates for GFP and GFP-8b from **b**. **p < 0.001 with Student’s *t*-test. N = number of transfected cells examined. **d** Immunoblots for indicated proteins from triton soluble and triton insoluble fractions of transiently transfected HeLa cells. Experiment repeated a minimum of three times with similar results

As previous studies have shown that ORF8b forms vesicle-like puncta in the cytosol, we used the bioinformatics aggregation prediction server PASTA2 (http://protein.bio.unipd.it/pasta2/)^20^ to investigate the intrinsic aggregation propensity of ORF8b. Analysis of the ORF8b sequence predicted a tendency to aggregate based on a carboxy-terminal VLVVL motif (aa 75–79), while re-analyzing the ORF8b sequence with V77 mutated to K (V77K) reduced its predicted propensity to aggregate (Fig. 1a). To empirically test the aggregation tendency of ORF8b, we expressed GFP-ORF8b (GFP-8b) in Hela cells overnight and imaged the transfected cells by confocal microscopy. While some cells had a homogenous pattern of expression, other GFP-8b expressing cells showed intracellular patch and fiber aggregation patterns (Fig. 1b). Quantification showed a considerably higher percentage of cells with intracellular aggregates in GFP-8b expressing cells than GFP vector (GFP) expressing cells (Fig. 1c). Presumably, the homogenous expression of GFP-8b precedes the development of intracellular aggregates. These findings were replicated in A549 cells, a human lung epithelial cell line (Fig. 1b).

Aggregated proteins often form detergent insoluble deposits. Thus, we separated the triton soluble and insoluble fractions of HeLa cells expressing either GFP, GFP-ORF8b, or mutant GFP-8b V77K to determine if ORF8b forms insoluble aggregates. To separate these fractions, we lysed the cells in a Triton-X based buffer and spun down the resultant lysates at 14,000 rpm for 10 min (min). The supernatant was collected and considered the triton soluble fraction, while the pellet was reconstituted in an LDS based buffer and heated at 100 ^°^C for 30 min. While preparing the triton insoluble fraction, we consistently observed that addition of LDS and heating for 30 min was unable to completely solubilize the pellet from GFP-8b expressing cells, but completely solubilized the pellet from GFP and GFP-8b V77K expressing cells. Accordingly, analysis of each fraction by SDS-PAGE followed by immunoblotting for GFP showed considerable monomeric and oligomerized GFP-8b in the insoluble fraction, while significantly less monomeric and almost no oligomerized protein was seen in the GFP-8b V77K insoluble fraction (Fig. 1d). The control GFP was undetectable in the insoluble fraction and seen only in the soluble fraction (Fig. 1d). Moreover, we observed consistently less GFP-8b V77K expression in the soluble fraction compared to GFP-8b, indicating that GFP-8b’s ability to aggregate may be protective against degradation (Fig. 1d).

**Fig. 2:**
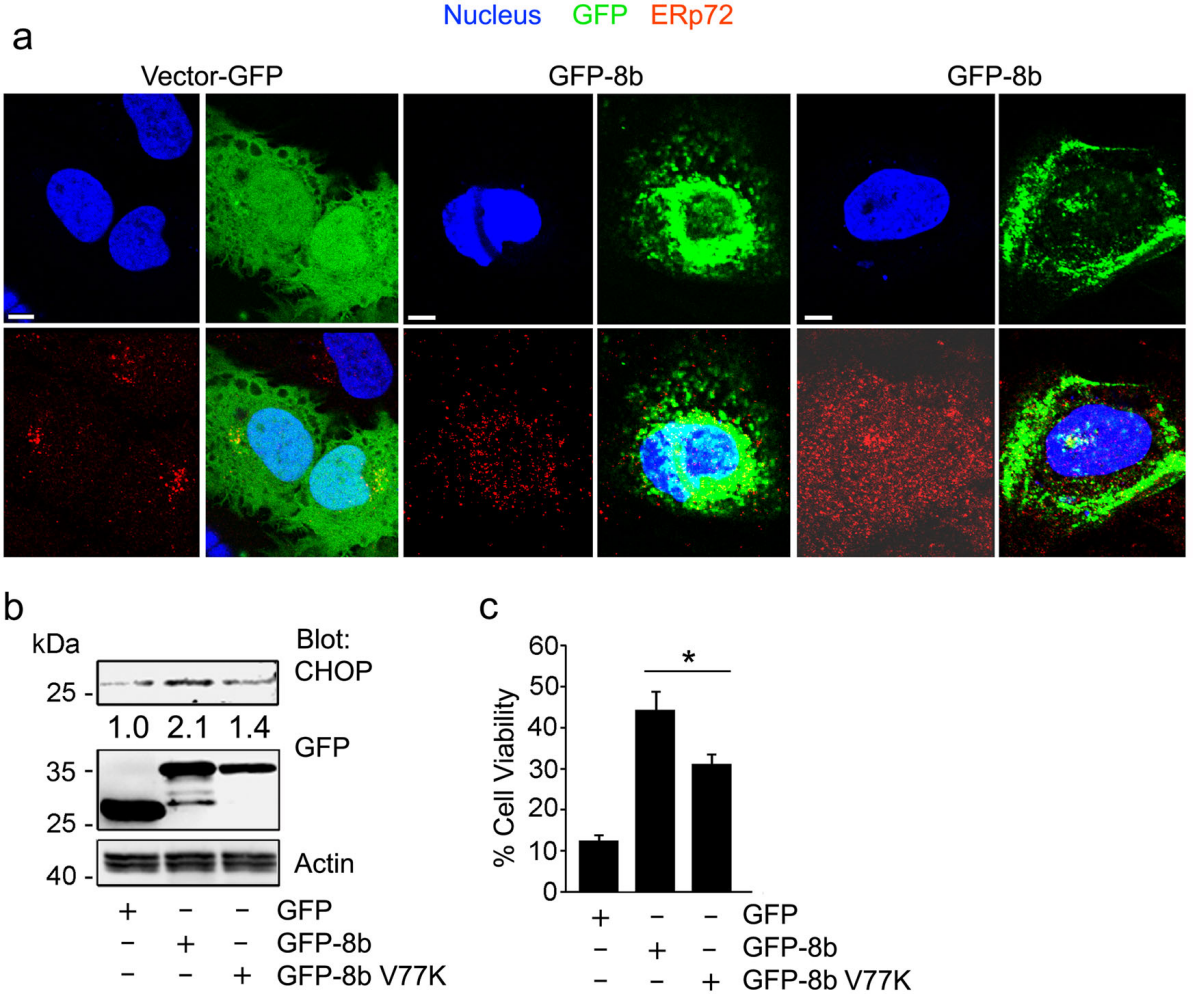
ORF8b induces ER stress and cell death. **a** Confocal microscopy images of HeLa cells transiently transfected with either GFP or GFP-8b and immunostained for the endoplasmic reticulum marker ERp72 (red). Nuclei were counterstained with DAPI and cells were fixed prior to imaging. Individual and merged images are shown, and the scale bar is 5 μm. **b** Immunoblot looking at the accumulation of the ER stress marker CHOP after transient transfection of GFP, GFP-8b, or GFP-8b V77K in HeLa cells. **c** Quantification of cell death by Tryptan Blue uptake after overnight transfection of HeLa cells with the indicated proteins. A minimum of three fields with >50 cells per field were quantified. Statistical significance evaluated with Student’s *t-*test (*p < 0.05, **p < 0.01). Each experiment repeated a minimum of three times

Intracellular protein aggregates and misfolded proteins cause ER stress and induction of the unfolded protein response (UPR), which can be quantified by detecting the upregulation of the UPR protein CHOP^21,22^. Confocal imaging showed that aggregated GFP-8b caused ER dispersion as assessed by immunostaining for ERp72 (Fig. 2a). The expression of GFP did not affect the usual juxta nuclear localization of the ER while expressing GFP-8b disrupted the usual ERp72 localization. Immunoblotting showed greater CHOP induction after transfection of GFP-8b than either GFP or GFP-8b V77K (Fig. 2b), indicating that GFP-8b forms protein aggregates dependent on its valine 77. As intracellular protein aggregation is toxic once the aggregates overwhelm cellular homeostatic mechanisms, we looked at cell death by Trypan blue uptake following expression of GFP, GFP-8b, and GFP-8b V77K. Expression of GFP-8b induced significantly more cell death than did GFP, while the GFP-8b V77K was partially protective (Fig. 2c). Taken together, this data suggests that GFP-8b forms insoluble protein aggregates and that can cause cell death, which at least partially depends on its ability to form intracellular aggregates.

### ORF8b induces lysosomal stress, the nuclear translocation of TFEB, and the activation of Transcription factor EB target genes

**Fig. 3:**
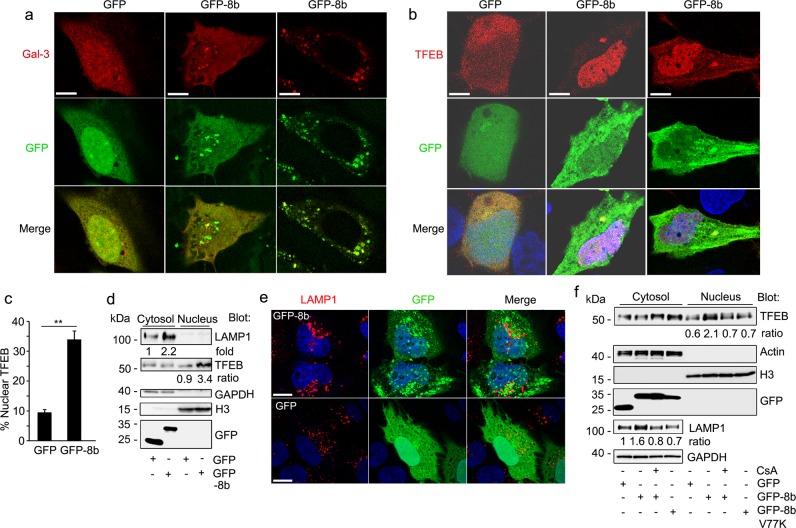
ORF8b triggers lysosomal stress and TFEB nuclear translocation. **a**, **b** HeLa cells were transiently transfected with the indicated plasmids. Confocal microscopy images of GFP or GFP-8b co-transfected with RFP-galectin 3 (a) or mCherry-TFEB (b). Individual and merged images are shown, and the scale bar is 5 μm. **c** The percentage of cells with predominantly nuclear TFEB from part b was quantified for each condition (three fields, minimum of 50 cells/field). **p < 0.01 with Student’s *t*-test. **d** Immunoblot to assess LAMP1 and TFEB expression in nuclear and cytosolic fractions separated by SDS-PAGE. HeLa cells were transiently transfected with GFP or GFP-8b. **e** Representative confocal images from HeLa cell expressing GFP or GFP-8b and immunostained for LAMP1 expression. Individual and merged images are shown, and the scale bar is 5 μm. **f** Immunoblot of cytosol and nuclear extracts from GFP, GFP-8b, or GFP-8b V77K transfected HeLa cells treated overnight with or without the calcineurin inhibitor cyclosporin A (CsA- 400 nM). Images and blots are representative data from experiments repeated a minimum of 3 times

Aggregated proteins are typically cleared from the cytosol by the autophagy–lysosome pathway, which engulfs bulk cargo in the cytoplasm and delivers it to the lysosome for degradation^23,24^. Formation of intracellular protein aggregates can disrupt this pathway, as aggregated proteins are poorly degraded by lysosomes. Lysosomal protein buildup then results in lysosomal stress and compensatory activation of transcription factor EB (TFEB), the master transcriptional regulator of autophagic and lysosomal machinery^25,26^. To determine if ORF8b disrupts autophagic and lysosomal homeostasis, we expressed GFP-8b and mCherry-galectin 3 in HeLa cells followed by imaging via confocal microscopy. Under homeostatic conditions galectin-3 is localized diffusely throughout the cytosol, but rapidly forms puncta on lysosomes following lysosomal stress/damage^27^. Accordingly, GFP expressing cells showed diffuse cytosolic galectin-3 distribution, while many GFP-8b expressing cells showed clear galectin-3 puncta formation, some of which co-localized with GFP-8b (Fig. 3a).

Correspondingly, expression of GFP-8b with mCherry-TFEB showed prominent TFEB nuclear localization compared to the control (Fig. 3b). Immunoblotting endogenous TFEB after separation of the nuclear and cytosolic fractions in GFP-8b expressing HeLa cells showed significantly more TFEB in the nucleus than control cells, and levels of the TFEB target gene LAMP1 were consistently increased (Fig. 3c). Confocal microscopy of GFP-8b expressing cells showed large, prominent lysosomes as assessed by LAMP1 immunofluorescence (Fig. 3d). However, we noted only moderate overlap between the LAMP1 immunostaining and the GFP-8b aggregates. TFEB nuclear translocation following both ER and lysosomal stress has been reported to depend upon dephosphorylation of its S211 residue by the phosphatase calcineurin (PPP3CB)^28–30^. To determine the mechanism of TFEB activation in GFP-8b overexpressing cells, we immunoblotted endogenous TFEB following nuclear fractionation of GFP, GFP-8b, and GFP-8b V77K expressing cells. The GFP-8b expressing cells were treated or not with the calcineurin inhibitor cyclosporin A. Again, GFP-8b induced TFEB nuclear translocation and increased LAMP1 expression, both of which were reduced by treatment with cyclosporin A. Expression of GFP-8b V77K did not increase nuclear TFEB levels or increase the level of LAMP1 in the cytosolic fraction (Fig. 3e). These data indicate that ORF8b aggregates causes ER and lysosomal stress, which leads to the nuclear translocation of TFEB, and the upregulation of TFEB target genes.

### ORF8b increases autophagy

**Fig. 4:**
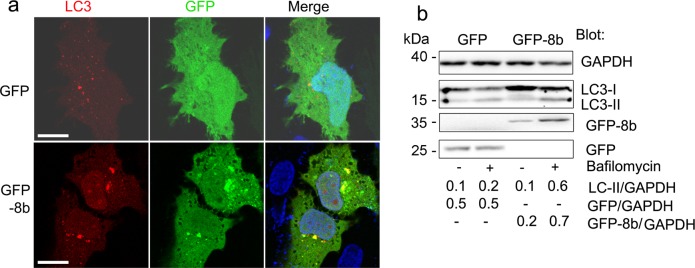
ORF8b enhances autophagic flux and autophagosome formation. **a** Confocal microscopy images of HeLa cells co-transfected with the autophagosome marker RFP-LC3 and either GFP or GFP-8b showing GFP-8b induced RFP-LC3 puncta and GFP-8b/RFP-LC3 co-localization. Individual and merged images are shown, and the scale bar is 5 μm. **b** Immunoblot of LC3 and the indicated proteins from whole cell lysates of GFP or GFP-8b transfected HeLa cells treated with or without Bafilomycin A1 (100 nM, 4 h). Band density quantification was normalized to GAPDH. Images and blots are representative data from experiments repeated a minimum of 3 times

Activation of TFEB increases both lysosomal biogenesis and autophagic flux^31^, thus we checked to see whether GFP-8b affects autophagy downstream of TFEB. Expression of GFP-8b induced clear formation of RFP-LC3 puncta, while GFP did not (Fig. 4a). In some cells the RFP-LC3 defined autophagosomes strongly co-localized with the GFP-8b aggregates while in other cells many of the aggregates did not (Fig. 4a). An increase in LC3 puncta can be due to an increase in autophagosome formation or a decrease in autophagosome/lysosome fusion^32^. During autophagosome formation, LC3 is lipidated and converted from LC3-I to LC3-II. We expressed GFP or GPF-8b in the presence or absence of Bafilomycin A1, which blocks autophagosome-lysosome fusion, and immunoblotted the cell lysates for LC3. We found that the cell lysates from the GFP-8b and GFP expressing cells had similar LC3-II levels. However, bafilomycin A1 treatment increased the LC3-II levels in the GFP-8b expressing cells more than it did in the control cells (Fig. 4b). These results indicate that GFP-8b induces autophagic flux. Treatment with bafilomycin A1 increased the GFP-8b levels, but not GFP levels suggesting that ORF8b aggregates are partially degraded by the autophagy–lysosome pathway. Taken together, the GFP-8b intracellular aggregates trigger cellular stress. This results in a calcineurin dependent activation of TFEB and TFEB target genes, which leads to an increase in autophagic flux.

### ORF8b expression triggers inflammasome activation in macrophages

**Fig. 5:**
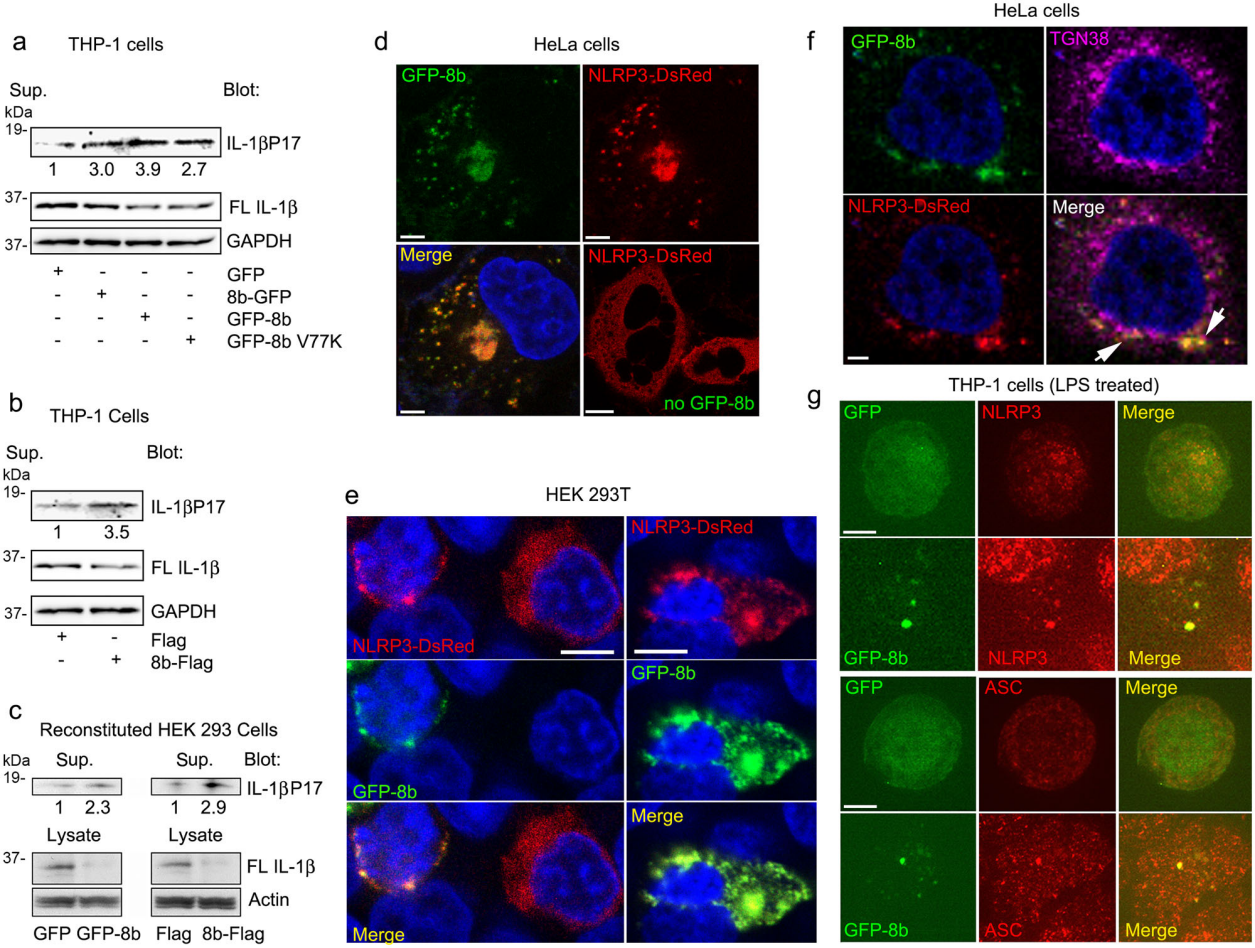
ORF8b triggers NLRP3 inflammasome activation. **a** Immunoblots for the indicated proteins of PMA differentiated THP-1 cell supernatant and lysates after transient transfection of expression vectors for GFP, 8b-GFP, GFP-8b, or GFP-8b V77K. **b** Immunoblots for the indicated proteins of PMA differentiated THP-1 cell supernatant and lysates after transient transfection of Flag or 8b-Flag. **c** Immunoblots for the indicated proteins of HEK 293T cell supernatants and lysates expressing the requisite inflammasome components and either GFP-8b or 8b-Flag with their appropriate control vectors. **d** Confocal microscopy images of HeLa cells expression NLRP3-DsRed only or NLRP3-DsRed and GFP-8b. Nuclei were counterstained with DAPI and cells were fixed prior to imaging. Individual and merged images are shown, and the scale bar is 5 μm. **e** Confocal microscopy images of HEK 293T cells expressing NLRP3-DsRed and GFP-8b. Left panels show co-transfected cell and a cell expressing NLRP3-DsRed alone. The right panels show a cell expressing both NLRP3-DsRed and GFP-8b. Individual and merged images are shown, and the scale bar is 5 μm. **f** Confocal microscopy images of HeLa cells expressing NLRP3-DsRed and GFP-8b and immunostained for TGN38. Individual and merged images are shown, and the scale bar is 3 μm. Images from a single confocal slice. Arrows point areas of overlapping signal. **g** Confocal microscopy images of THP-1 cells LPS activated and transfected with either GFP or GFP-8b. The following day the cells were immunostained for NLRP3 (upper panels) or ASC (lower panels). Individual and merged images are shown, and the scale bar is 5 μm. Images and blots are representative data from experiments repeated a minimum of 3 times

Intracellular protein aggregation and lysosomal stress have previously been mechanistically linked to NLRP3 activation^33,34^. As SARS patients show increased serum IL-1β and IL-18^7,8^, we investigated whether ORF8b affects inflammasome activation in macrophages. To do so, we first attempted to generate a permanent cell line expressing GFP-8b in the human macrophage cell line THP-1 cells. While we easily established GFP expressing THP-1 cells, no cells survived the selection process when we attempted to express GFP-8b. This is likely secondary to the toxicity of the protein, as even attempts at using an inducible promoter failed to generate a useable cell line. Therefore, we switched to a transient transfection system. Evaluating inflammasome activation in macrophage cell lines following transient DNA transfection can be problematic as cytosolic DNA activates DNA sensing inflammasomes. However, we found that that by carefully titering the amount of DNA transfected, we could express the GFP vector and yet trigger minimal Il-1β production. Therefore, we compared IL-1β production by THP-1 cells transiently transfected with low amounts of the vectors producing GFP, GFP-8b, 8b-GFP, or GFP-8b V77K. We found that both GFP-8b and 8b-GFP increased the amount of IL-1β present in the cell supernatant compared to GFP alone (Fig. 5a). Surprisingly, the GFP-8b V77K construct also enhanced IL-1β production suggesting that the mechanism did not solely depend upon lysosomal stress (Fig. 5a). We also checked whether an alternatively tagged version could trigger IL-1β production by expressing Flag tagged version of ORF8b. Similar to the results with the GFP-tagged ORF8b, we found that 8b-Flag induced 3-fold more Il-1β than did the Flag vector (Fig. 5b). A complementary approach to assess inflammasome activity is to reconstitute the requisite components in HEK293T cells. Therefore, we transfected HEK293T cells with expression vectors for NLRP3, caspase-1, ASC, and full-length IL-1β in the presence of GFP or GFP-8b. A similar experiment was performed using FLAG or 8b-FLAG. In both instances we found an increase in IL-1β in the supernatant and a decrease in full-length IL-1β in the cell lysate in the presence of the ORF8b constructs versus the controls (Fig. 5c). These results suggest that ORF8b activates NLRP3 inflammasomes in macrophages and likely in monocytes.

In NLRP3 expressing cells, signals that activate the NLRP3 inflammasomes cause the disassembly of the trans-Golgi network (TGN) and the recruitment of NLRP3 to the dispersed TGN, which serves as a scaffold for NLRP3 aggregation^35^. This leads to the polymerization of the adapter protein ASC and recruitment of caspase-1. To assess the impact of GFP-8b on the localization of NLRP3, we expressed NLRP3-DsRed in HeLa cells in the presence or absence of GFP-8b. In the absence of GFP-8b NLRP3 expressed homogenously throughout the cytosol of transfected HeLa cells, however the presence of GFP-8b dramatically altered NLRP3 localization (Fig. 5d). We found that both proteins co-localized in the cytosol in small cytoplasmic dots as well as in a large perinuclear aggregate. We performed a similar experiment using HEK 293T cells (Fig. 5e). In the left panel the images show a HEK 293T cell expressing NLRP3 alone and one expressing both proteins, while the right panel shows another cell co-expressing NLRP3 and GFP-8b. We found that GFP-8b forms large aggregates in HEK 293T cells and that its expression disturbed the homogenous cytosolic pattern of NLRP3, and as we had observed with HeLa cells both proteins showed an overlapping expression pattern. We also checked the distribution of the trans-Golgi network in HeLa cells co-expressing GFP-8b and NLRP3-DsRed by immunostaining for TGN38 (Fig. 5f). We found partial co-localization between the NLRP3-DsRed and GFP-8b cytoplasmic dots and TGN38.

In macrophages, NLRP3 also adopts a homogenous pattern until activating signals rapidly trigger the dispersion of the TGN and recruitment of NLRP3 in a punctate pattern. As macrophages also express ASC, the punctate NLRP3 distribution rapidly redistributes into a single speck that contains NLRP3 and ASC. When we expressed GFP-8b in LPS activated THP-1 cells we found that a major portion of the GFP-8b localized in a single speck that co-localized with endogenous NLRP3 and ASC (Fig. 5g). These results also suggested that ORF8b targets NLRP3.

### ORF8b may directly target NLRP3 by binding to its LRR domain

**Fig. 6:**
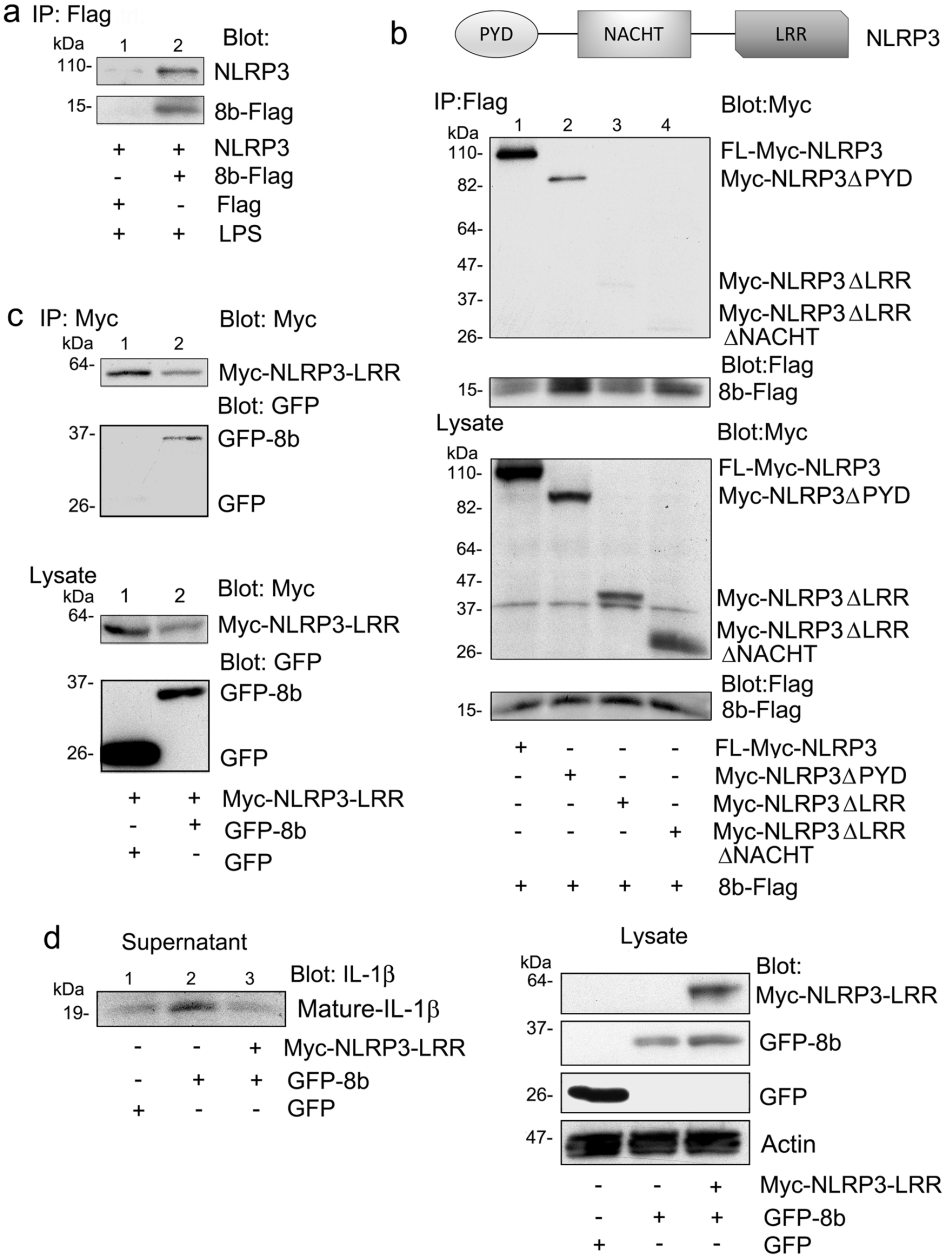
ORF8b interacts with the LRR domain of NLRP3. **a** Endogenous NLRP3 immunoblot of Flag immunoprecipitates using cell lysates prepared from THP-1 cells transiently expressing 8b-Flag. The cells were transfected the day prior to use and treated with LPS (50 ng/ml) overnight. **b** Myc or Flag immunoblot of Flag immunoprecipitates (top) or cell lysates (bottom) prepared from HEK 293 T cells expressing the indicated Myc tagged NLRP3 constructs and 8b-Flag. A schematic representation of the NLRP3 domain organization is shown for clarity. **c** Myc or GFP immunoblots of Myc immunoprecipitates and cell lysates from HEK 293 cells expressing Myc-NLRP3-LRR and either GFP or GFP-8b. **d** Indicated immunoblots of cell supernatant and lysates from HEK 293 T cells expressing NLRP3, Casp-1, ASC, full-length IL-1β, and Myc-NLRP3-LRR in the presence of GFP or GFP-8b. Images and blots are representative data from experiments repeated a minimum of 3 times

Enterovirus 71 (EV71) 3D protein stimulates NLRP3 inflammasome activation by interacting with NLRP3 to facilitate inflammasome complex assembly. EV71 3D interacted with NLRP3 through the NACHT and LRR domains. To test whether ORF8b might also interact with NLRP3, we transiently expressed 8b-Flag in PMA differentiated and LPS stimulated THP-1 cells and tested whether we could detect an association with endogenous NLRP3. We found that immunoprecipitated 8b-Flag pulled down endogenous NLRP3, while control immunoprecipitates did not (Fig. 6a). To characterize the region in NLRP3 domain responsible for the interaction, we examined the binding of Myc-tagged truncated NLRP3 proteins to 8b-Flag. In addition to full-length NLRP3, we used constructs that express NLRP3 with a deleted leucine-rich repeat (LRR) domain (NLRP3ΔLRR), a deleted Pyrin domain (NLRP3ΔPYD), or deleted NACHT and LRR domains (NLRP3ΔLRRΔNACHT). Following transient expression, 8b-Flag interacted with both full-length NLRP3 and the PYD domain deleted protein, but not the LRR and LRR/NACHT domains deleted proteins (Fig. 6b) To directly show that ORF8b interacts with the LRR domain of NLRP3, we expressed the NLRP3 LRR domain as a Myc tagged protein (amino acids 537–991) and collected Myc immunoprecipitates following GFP-8b or GFP expression. We found GFP-8b, but not GFP in the Myc-immunoprecipitates (Fig. 6c). Next, we expressed the non-functional NLRP3 LRR domain and determined whether its presence affected the induction of mature IL-1β secretion by ORF8b. We found that expression of the NLRP3 LRR domain impaired the production of mature IL-1β after GFP-8b expression in inflammasome reconstituted HEK 293T cells (Fig. 5e), confirming the functional importance of this interaction. In summary, our data indicates that ORF8b activates the NLRP3 inflammasome and directly targets the NRLP3 LRR domain.

## Discussion

Though the exact mechanistic role of ORF8b in SARS-CoV pathogenesis remains unclear, it is well established that the splitting of ORF8 corresponds with the shift from the early to the middle stage of the SARS epidemic, with the majority of affected patients being infected by virus containing the split ORF^36^. Recent advances in mouse models have shown that SARS pathogenesis is driven by high initial virus titers resulting from a late interferon response, which drives aberrant recruitment of IMMs and activation of the innate immune response resulting in cytotoxicity^9^. Given the likelihood that ORF8b is evolutionarily adaptive for the virus^10^, it follows that ORF8b may contribute to SARS-CoV pathogenesis by impairing the host interferon response and/or targeting the innate immune system. While the former has been previously shown^13^, here we show that ORF8b activates intracellular stress pathways upon forming insoluble aggregates and directly targets innate immunity by activation of the NLRP3 inflammasome.

Intracellular protein aggregation contributes to the pathogenesis of a variety of diseases and is both propagated by and contributes to inflammation^37,38^. Protein aggregates have long been observed after viral infection; viruses are known to use aggregates as scaffolds for replication and/or for shielding from host degradation^39^. In our study, ORF8b appears to be degraded by the autophagy–lysosome system, as treatment with bafilomycin A1 increases ORF8b levels. The aggregation ability of ORF8b may be protective against degradation, as we consistently observed that the anti-aggregation V77K mutant is expressed at lower levels than ORF8b. It has previously been reported that ORF8ab and ORF8b induce ER stress^40^, which is consistent with our findings showing accumulation of CHOP by ORF8b expression dependent on V77. We also find induction of lysosomal stress and activation of TFEB by ORF8b. Both ER stress and TFEB have been shown to propagate the inflammatory response, with TFEB directly promoting the transcription of inflammatory cytokines^26,41,42^. This may be a cellular mechanism by which ORF8b activates the innate immune response in macrophages and contributes to SARS-CoV pathogenesis.

Finally, RNA or DNA virus infection is known to trigger NLRP3 or AIM2 inflammasome activation^43,44^. The released IL-1β and IL-18 functions to promote host defense, but aberrant inflammasome activation can result in excessive inflammation and enhanced disease. Examples include the influenza encoded proton-selective ion channel M2 which provides signal 2 for NLRP3 inflammasome activation^45^, the encephalomyocarditis virus viroporin 2B which promotes the release of intracellular Ca^2+^ from intracellular stores also providing signal 2^46^, and the pathogenic influenza A viruses which use PB1-F2 to activate NLRP3 inflammasomes^47^. Our findings indicate the SARS-CoV can activate NLRP3 inflammasome in macrophages via ORF8b. While SARS-CoV abortively infects macrophages/monocytes, enough ORF8b may be present to impact lysosome integrity, autophagy pathways, and NLRP3 inflammasomes. In contrast to macrophages, the SARS-CoV productively replicates in lung epithelial cells. These cells also express NLRP3 and can assemble NLRP3 inflammasomes. In humans infected with the SARS-CoV the full impact of ORF-8b on the pathways we delineated in this study are likely in the lung epithelium. ORF8b may contribute to the cytokine storm and inflammasome activation that occurs during severe SARS-CoV infection. Moving forward, live virus deletion studies are required to assess the effects of ORF8b mediated intracellular aggregates and ORF8b mediated NLRP3 activation. However, here we identify novel mechanisms through which ORF8b may contribute to SARS pathogenesis.

## Material and methods

### Reagents and Abs

The following primary Abs were used for immunoblotting: NLRP3 (Cell Signaling 13158, 1:1000), IL-1β (Cell Signaling 12703, 1:1000), GFP (Cell Signaling 2955, 1:1000), TFEB (Cell Signaling 4240, 1:1000), CHOP (Cell Signaling 2895, 1:1000), LC3A/B (Cell Signaling clone D3U4C, 1:1000) Histone H3 (Cell Signaling clone D1H2, 1:2000), Myc (EMD Millipore clone 9E10, 1:2000), Flag (Sigma F1804, 1:1000), ASC (Santa Cruz sc-22514, 1:1000), LAMP1 (Santa Cruz clone H3A4, 1:2000), GAPDH-HRP (ProteinTech HRP-60004, 1:5000), and actin-HRP (Sigma A3854, 1:10,000). Anti-mouse (Cell Signaling 7076) or anti-rabbit (Cell Signaling 7074) HRP-conjugated secondary antibodies were used for ECL based detection. Primary Abs for immunofluorescence were NLRP3 (Enzo AlX-804-819), ASC (Santa Cruz sc-22514), ERp72 (Cell Signaling D70D12), and TGN38 (Novus, NBP1-03495). Alexa 568 conjugated polyclonal anti-mouse (Thermo Fisher, A11004) and anti-rabbit (Thermo Fisher, A11011) were used as secondary Abs prior to fluorescent imaging. For immunoprecipitation experiments, we used GFP antibodies coupled to magnetic beads (MBL, D153-9) and Flag antibodies coupled to agarose (Sigma, A2220) to pull down tagged proteins prior to immunoblotting. Cyclosporin A (R&D Systems, 1101) and Z-VAD-FMK (Sigma, V116) were used at 10 μM overnight, and Bafilomycin A1 (Sigma, B1793) at 100 nM for 4 h.

### Cells, plasmids, and siRNAs

THP-1, HEK293, A549, and HeLa cells were obtained from the American Type Culture Collection (ATCC) and maintained following ATCC’s recommendations. THP-1 monocytes were differentiated into macrophages by treating with PMA (50 nM) for 3 h. To generate stable THP-1 cell lines, constructs expressing 8b-GFP or GFP were transfected followed by G418 (200 μg/ml) selection for 6 weeks. GFP-positive cells were FACS sorted twice and expanded. SARS-CoV ORF8b cDNA was generated by PCR from K14 and J17 cDNA clones by Dr. H.Y. Qi and Dr. James Shelhamer (Critical Care Medicine Department, National Institutes of Health), which were produced from the SARS-CoV genome Tor2 isolate (Michael Smith Genome Sciences Centre, Vancouver, Canada)^48^. PCR primers for cDNA generation were derived from the SARS-CoV accessory gene sequences in accession number NC_004718 (NCBI). Constructs expressing N and C terminal GFP-tagged SARS-CoV ORF8b were made using the pEGFP-N1 and C1 vectors (Clontech). PCR mutagenesis of GFP-8b was used to generate the V77K GFP-8b point mutant, and ORF8b-Flag was made by replacing the 8b-GFP c-terminal GFP with a 3x Flag tag. All constructs were verified by DNA sequencing.

Myc tagged NLRP3 plasmids were kindly provided by Dr. Yong-Jun Liu (Baylor). The Myc tagged NLRP3 LRR domain construct spanned amino acids 537–991 in NLRP3. The pcDNA3 NLRP3-Flag construct was kindly provided by Dr. Gabriel Nunez (University of Michigan). To construct the NLRP3 Ds-Red construct, the Ds-Red coding region was PCRed from the pDs-Red-Monomer fluorescent vector (Clontech) and inserted into Flag tagged NLRP3 construct after removal of the c-terminal Flag coding sequence. The TFEB-GFP plasmid was bought from Addgene, (38119) and the TFEB-mCherry plasmid made by moving the TFEB cDNA into the mCherry N1 vector (ClonTech). The ptf-Galectin 3 plasmid was also purchased from Addgene (64149), and the mCherry-Galectin 3 plasmid made by removing the GFP from the ptf-Galectin 3 vector. Pooled siRNAs targeting NLRP3 (sc-45469), ASC (sc-37281), GFP (sc-45924), and a scrambled control (sc-37007) were purchased from Santa Cruz Biotechnology. Plasmids and siRNA were transfected into cells using X-tremeGENE-HP (Roche, 6366236001) following the manufacture’s protocol.

### Immunoblot analysis and immunoprecipitation

For standard immunoblotting, cells were lysed in Buffer A containing 20 mM HEPES (pH 7.4), 50 mM β-glycerophosphate, 1% (v/v) Triton X-100, 2 mM EGTA, and cOmplete protease inhibitor cocktail (Sigma, 11836170001) plus PhosStop (Sigma, 04906837001) phosphatase inhibitor tablets for 30 min. Lysates were cleared by centrifugation at 14,000 rpm for 10 min and the supernatant collected. For separation of Triton soluble and insoluble fractions, cells were lysed in Buffer A and centrifuged as before. The supernatant was considered the Triton soluble fraction, while the pellet was directly mixed with 1x NuPage LDS Sample Buffer (Invitrogen, NP0008) and heated at 100 °C for 30 min (Triton insoluble fraction). For nuclear fractionation, cells were lysed with Buffer B, which is Buffer A substituting 0.5 (v/v) NP-40 for Triton X-100. After centrifugation, the supernatant was considered the cytosolic fraction. The resultant pellet was washed 4–6 times with Buffer B and then lysed in Buffer B + 0.5% (v/v) SDS for 30 min, this was considered the nuclear fraction. Inflammasome activation was measured by immunoblotting cell culture supernatants for mature IL-1β and cleaved caspase-1 as previously described^49^. For immunoprecipitations, cells were lysed with Buffer C, which is Buffer A + 0.5% (w/v) CHAPS and incubated with either GFP or FLAG beads for 3 h. Immunoprecipitates were washed 4–6 times and resuspended in 1x NuPage LDS buffer to release captured proteins prior to immunoblotting.

All lysates were mixed with 4x NuPage LDS Sample Buffer and heated at 100 °C for 10 min, followed by separation on a 4–20% Tris-Gylcine Gel (Invitrogen) and transfer to a nitrocellulose membrane using the iBlot Gel Transfer System (Invitrogen). The membrane was blocked with 5% nonfat milk (or 5% BSA) in TBST (25 mM Tris-HCl, 150 mM NaCl, 0.1% Tween-20) for 1 h and incubated at 4 °C overnight with the primary Ab in TBST with 5% BSA. The appropriate secondary Abs conjugated to HRP were used to detect the protein of interest via ECL. When necessary, membranes were stripped using Restore Plus Western Blot Stripping Buffer (Thermo, 46430) following the manufacturers protocol, re-blocked, and reblotted. Images were acquired either by exposure on film (Amersham Hyperfilm ECL) or using the iBright 1000FL (Invitrogen). Blots were scanned and imported into Photoshop as unmodified tagged image files; quantification of band intensity was performed using standard methods on ImageJ (NIH) and presented relative to the appropriate housekeeping gene.

### Immunofluorescence

THP-1 cells transiently transfected to express GFP-8b or GFP were seeded on glass-bottom collagen coated microwell dishes (MatTek), differentiated with PMA, and incubated overnight with LPS (50 ng/ml) and a caspase-1 inhibitor (Z-VAD-FMK, 10 μM). The following day, the cells were washed, fixed with 4% paraformaldehyde, permeabilized with 0.1% Triton X-100 in PBS, and blocked with 1% bovine serum albumin for 15 min. For immunostaining, the cells were incubated for 4 h at room temperature or overnight at 4 °C with primary antibodies diluted in 1% bovine serum albumin/PBS. Fluorescent images were collected after secondary antibody incubation using a Leica TCS-SP5-X-WLL confocal microscope equipped with an argon and white light laser (Leica Microsystems) at either 63X or 100X oil immersion objective (NA 1.4). The samples were excited with 488- or 561-nm laser lines. Image analysis was performed using Imaris 8.0.0 (Bitplane AG) and Adobe Photoshop CS3 (Adobe Systems). HeLa cells transiently transfected to express NLRP3-Ds-Red plus or minus GFP-8b were washed 6 h after transfection and plated in glass-bottom microwell dishes overnight. Plated cells were processed and imaged as outlined above the following day. For DNA staining, the cells were incubated with 30 nM DAPI (Sigma) in PBS for 30 min and washed prior to imaging.

### NLRP3 inflammasomes reconstitution

HEK293 cells were seeded onto 24-well plates in standard media. The following day, the media was changed to OPTI-MEM (Gibco) and the cells transfected with constructs expressing NLRP3 Flag (15 ng), Casp-1 Flag (5 ng), ASC (5 ng), full-length IL-1β Flag (150 ng) and either a control plasmid (GFP or Flag) or GFP-8b or 8b-Flag. Cell supernatants and lysates were immunoblotted for full-length and mature IL-1β the next day.

### Cell death assay

0.4% Tryptan Blue (Sigma T8154) was added to cell culture plates and incubated at room temperature for 2 min. Percentage of non-viable cells (cells that had taken up Tryptan Blue) was determined manually by light microscopy and recorded; a minimum of three fields with >50 cells per field were quantified.

### Statistics

All immunoblots, and immunofluorescence images shown are representative of reproducible experiments. Differences between groups were determined using Student’s *t*-test (*p* < 0.05 was considered significant), and all statistical analysis was performed on GraphPad Prism 6.0. All experiments were performed a minimum of three times.
